# Design and practice of innovative practice workshop for new nurses based on creativity component theory and outcome based education(OBE) concept

**DOI:** 10.1186/s12909-023-04684-5

**Published:** 2023-09-26

**Authors:** Hao Yang, Hui Zhu, Wanying Luo, Wentao Peng

**Affiliations:** 1 Department of Medical Genetics Nursing, West China Second University Hospital, Sichuan University/West China School of Nursing, Sichuan University, Chengdu, Sichuan China; 2 Key Laboratory of Birth Defects and Related Diseases of Women and Children (Sichuan University), Ministry of Education, Chengdu, Sichuan China; 3 Department of Nursing, West China Second University Hospital/West China School of Nursing, Sichuan University, Sichuan University, Chengdu, Sichuan China

**Keywords:** Outcome based education(OBE), Creative ability component theory, Innovative practice, Workshop, Practice

## Abstract

**Objective:**

To design a teaching model of innovative nursing practice workshop for new nurses based on creativity component theory and OBE concept, and to explore its implementation effect and application evaluation.

**Methods:**

Using convenience sampling, 50 newly recruited nurses in 2021 from a tertiary hospital in Chengdu were selected as the study subjects and taught using the new nurses’ innovative practice workshop based on creativity component theory and the OBE concept.

**Results:**

Before and after the implementation of the teaching, the new nurses’ creativity scale scores were significantly improved, and the effects of practice demonstration, teaching satisfaction results, and research output (one-year follow-up) were better. All 50 new nurses (100%) expressed willingness to participate in the course again.

**Conclusions:**

Based on creativity component theory and the OBE concept, the innovative practice workshop for new nurses integrates theory and practice, and fully mobilizes students’ thinking, interest, and subjective initiative; during the teaching process, students’ creative thinking and problem-solving skills are improved, in addition, teamwork, literature review, communication and other skills are improved to different degrees, which is conducive to the research results. In addition, students’ abilities in teamwork, literature review, communication, and other aspects have been improved to different degrees, which is conducive to producing scientific research results and lays a good foundation for their future career development.

**No patient or public contribution:**

No patient or public contribution is involved in this study.

## Introduction

Compared to Western countries, the evolution of higher nursing education in China began relatively recently, with the education and training of postgraduate nursing students commencing approximately 20 years ago. Consequently, the maturation of elite nursing personnel training in China remains in its nascent stages. A significant portion of the current nursing workforce in China possesses a lower educational background. This is evidenced by a notable scarcity of highly-educated nursing talents, a limited foundation in scientific research, and a pronounced deficiency in innovative consciousness [1]. In a study by Lu Han [2], which assessed the relationship between innovation capacity and general self-efficacy among 132 undergraduate nursing students in a Shandong-based university, it was found that their innovative ability was moderately low. Similarly, Yang Li’s research [3], evaluating the innovative behavior and its determinants among 1,386 clinical nurses, indicated that their creative behavior was average.

Innovation serves as an indispensable catalyst for the sustained growth of any discipline and is pivotal for societal progress. As emphasized in the report of the 19th National Congress, the repeated mention of “innovation” underscores its quintessential role in national development. The emphasis on innovation is evident, emphasizing its significance not only at a national level but also for the advancement of specific disciplines, particularly in the rapidly evolving medical field, which in turn influences its trajectory and pace. Thus, strategically nurturing innovation skills within nursing and enhancing the profession’s core competitiveness is paramount for the discipline’s growth and the broader advancement of nursing technology innovation. In the realm of educational reform, the essence of innovative practice is to refine the educational process and more effectively cultivate innovative talents. Consequently, optimizing this innovative practice becomes imperative.

Outcomes-Based Education (OBE) is a student-centered educational philosophy initially introduced by Spady [4]. OBE emphasizes the primacy of learning outcomes, advocating for a reverse-design approach to teaching and learning. Instead of traditional teacher-centered methodologies, it promotes student-centric cooperative and inquiry-based active learning [5]. This OBE philosophy has garnered significant attention and application in educational research both domestically and internationally [6–10]. Its roots can be traced back to behaviorism and cognitive psychology of the early 20th century. Behaviorists contend that behaviors arise from a stimulus-response mechanism, emphasizing the significance of reinforcement and punitive measures in shaping behaviors. In an educational context, this translates to teachers strategically designing stimuli to direct student behavior, reinforced or attenuated as required. Contrarily, cognitive psychology underscores the learner’s cognitive processes, viewing learning as an intricate process of contemplation and exploration rather than a mere reaction to external triggers [8–10]. This deviates from traditional higher education paradigms where students were passive recipients of knowledge. Instead, OBE accentuates active student participation, fostering self-assessment and enabling students to steer and self-regulate their learning [7]. By emphasizing the goals and outcomes of student learning [5], OBE offers a definitive purpose for pedagogical reforms. It is aligned with the objectives of equipping students with the requisite knowledge, skills, and attributes they will need upon integration into society, and is pivotal for nurturing multifaceted, application-oriented talents.

Introduced by German architect Walter Gropius in 1919, the workshop model was pioneered by the Staatliches Bauhaus. This teaching approach seamlessly combined “basic courses” with “workshop training”. Today, the workshop teaching method emphasizes communication and collaboration among participants, facilitating an integration of theory and practice through experiential activities such as participation, interaction, and discussion. This method effectively bridges the gap between theoretical knowledge and practical application in teaching [11]. It has garnered widespread adoption in fields including education, medicine, and nursing [12–17]. Existing literature indicates that workshop teaching not only enhances student engagement and teaching effectiveness [18] ,but also fosters the development of leadership, creativity [19], and pedagogical skills [15]. During the workshop, instructors provide the research topic and direction. Under their guidance, students collaborate in teams to undertake the research project, analyzing the problem comprehensively and identifying pivotal solutions. In this pedagogical approach, there’s a strong emphasis on leveraging each participant’s theoretical knowledge and practical skills, thereby maximizing the utility of both explicit and tacit knowledge.

The Creativity Component Theory, first introduced by scholar Amabile in 1983, is rooted in social psychology [18]. This theory posits that the creative process involves both personal and external environmental factors, encompassing domain-related skills, creativity-related strategies, and task-related motivation within the emotional realm [19]. Intrinsically or extrinsically derived, motivation propels individual creativity. However, possessing domain-related knowledge and skills alone does not guarantee creative outcomes; a strong motivational drive is also essential [20]. Moreover, individuals exhibit higher levels of creativity when they perceive their environment as positive [21].

**Fig. 1:**
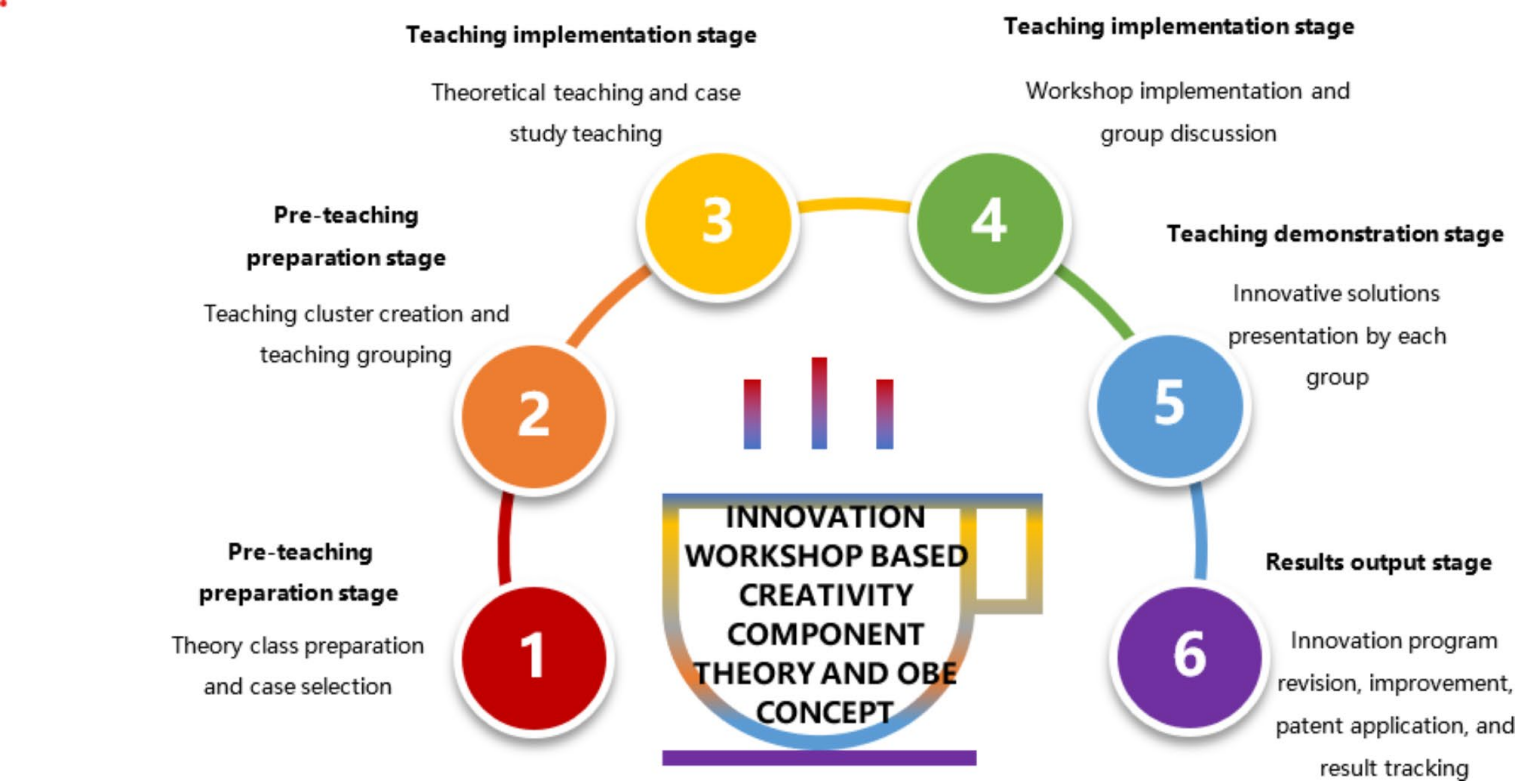
Innovation workshop for new nurses based on creativity component theory and outcome based education(OBE) concept

In light of the aforementioned theoretical and practical contexts, this study endeavors to synergize the Creativity Component Theory with an outcomes-based workshop model. Our aim is to enhance the pedagogical impact, fostering innovative thinking and practices within nursing. Consequently, we devised and tested a nursing creative practice workshop model under the OBE (Outcomes-Based Education) framework, with the positive results presented subsequently. Figure 1 illustrates the six teaching stages of the innovation workshop tailored for new nurses, drawing from both the Creativity Component Theory and the OBE concept.

## Data and methods

### Study population

A convenience sample was used to select 50 new nurses of a tertiary hospital in Chengdu City, Class 2021 as the study population, none of the participants had previously participated in innovation-related training. The new nurses’ innovation practice workshop was based on creativity component theory and the OBE concept was used for teaching.

### Establishment of an innovative teaching team

The team consisted of nursing and laboratory technicians, totaling 9 people. Among them, 3 are professors, 3 are associate professors, and 3 are registered nurses. The teachers responsible for classroom teaching should have rich experience in innovative practice (①patent authorization ≥ 3; ② paper publication ≥ 3; ③ in charge of research project topics ≥ 1). A group meeting was held one month before the start of the course to construct a preliminary teaching program plan based on the original “Innovation and Patent Application” course, and to clarify the teaching contents, methods, and forms of this workshop.

### Research methodology

The design of this course is based on the theory of “components of creativity” proposed by the contemporary American psychologist and creator Teresa M. Amabile [18] in 1983 and integrates the five stages of creativity: questioning, preparation, practice, verification, and evaluation. Based on the skills of the participants (providing materials and methods for the whole innovation process), the course incorporates theoretical knowledge points supported by innovation-related skills (organizing and planning the practice, etc.) and motivation (identifying problems, generating them, and applying them in practice, etc.). The teaching design of nursing innovation practice workshop shown as Table 1.

#### Questioning and preparation stage: teaching group establishment and grouping

Each group consists of 5–6 members, and each group elects a group leader on a voluntary basis, who was responsible for organizing the group members to participate in the teaching activities and helping to follow up on the progress of the group’s follow-up project. Finally, the teacher issued the pre-class thinking task and used Internet resources such as the Sichuan University library, search websites, and patent search websites to conduct relevant searches, and the group leader organized the group members to conduct online thinking and discussion.

#### Preparation stage: theoretical teaching and case lecture

The theoretical lecture phase consists of two parts: theoretical teaching and case lecture. In the theoretical teaching session, on the one hand, the participants are taught the overview and methods of innovation to help them build up an understanding of innovation and establish an awareness of innovation; on the other hand, the participants are introduced to the concept, role, and implementation of patents, etc., and they are guided by concrete patent results to understand that patents are a form of existence of innovation and an important component of scientific research results, and to help them build up the ability to turn ideas into results through the innovation path, from abstract to concrete, and form the final scientific research results.

In the case study session, we use the authorized patent as a case study and analyze the background technology, concrete implementation, and innovation. The teaching format is based on theory, case study, innovative thinking, and results-oriented, which deepens the participants’ understanding of theoretical and conceptual knowledge, deepens their internalization and understanding of knowledge, thus achieving the purpose of establishing innovative consciousness and forming innovative thinking, and laying the foundation of cognition and thinking for the subsequent practical sessions of the workshop.

#### Practice and validation stage: workshop practice

After the theoretical teaching, each group began to discuss within the group, in the process of problem presentation-problem analysis-problem solution-case formation-case summary, firstly, the teacher threw out the question: “In your usual clinical work or operation, have you encountered some uncomfortable and inconvenient situations; what methods will you adopt for optimization and improvement?“. Each group member actively expresses their ideas and forms a viewpoint idea by briefly stating the background, and the problems that exist, analyzing the causes that arise, and proposing solution measures; the best idea is selected as the implementation direction of the group; then brainstorming is conducted for that direction: the innovation composition, function, innovation point, actual value (social value, economic value) and other aspects. During the process, if you encounter any problems related to feasibility and lecture content, you can communicate and discuss with the teacher; for groups with low initiative and slow progress, the teacher will understand the reasons and guide the intervention in time. After completing the discussion, according to the framework content of the “innovative proposal” (including name, technical field, background technology, purpose, technical solution, beneficial effect, innovation point, social value, economic value, and attached figure), complete the first draft of the “innovative proposal”, and Prepare for the case presentation session.

#### Evaluation stage: presentation of each group’s case and critique

Each group leader will be responsible for the presentation of 5–10 min, including background, objectives, improvement/solution, beneficial effects, innovation points, social benefits, economic benefits and sketch, and division of work within the group. The teacher will grade and evaluate the presentation in terms of content form, information retrieval use, language expression, teamwork, etc.

#### Continuation and output stage: continuation and refinement and proposal submission

In this phase, the teacher acts as a mentor to start a year-long mentoring and results tracking, guiding each group’s innovative project, and giving process tracking and necessary assistance throughout. The team leader of each group is responsible for organizing the team members in the classroom, and in response to the teacher’s suggestions, dividing the work among them, organizing them to review the data and patent search, etc., and then brainstorming again within the group to revise the “innovation plan” and improve the innovation, practicality, scientificity, and operability of the plan. After that, the team leader and the patent staff revised and improved the “nursing innovation plan” again, and then formed the “patent application” and submitted it to the National Patent Office for patent application.

### Evaluation methods

#### General information questionnaire

A self-administered questionnaire was used to collect general information about the participants of this teaching activity, including age, gender, grade, education, place of residence, department, and other relevant information.

#### Creativity scale

The creativity scale was adapted, scale was selected by research scholar Meng Yi [22] based on the scholars’ definition of creativity based on the academic community, and six items were selected to form the creativity scale [23, 24], and its reliability Cronbach’s α was 0.933. In this study, the questionnaire reliability Cronbach’s α was 0.910, which indicates that the reliability of the questionnaire is good.

#### Teaching satisfaction

A self-administered teaching satisfaction questionnaire was used to measure teaching satisfaction to understand the participants’ satisfaction with this teaching activity, where teaching satisfaction = (number of very satisfying + number of satisfying) / total number of cases × 100%.

#### Demonstration practice assessment

Each group leader will be responsible for the presentation of the group’s innovative program, and each group will report for 3–5 min. The assessment includes scientificity (15 points), innovation (15 points), practicality (15 points), operability (15 points), presentation content and form (20 points), information retrieval and application (10 points), language expression (5 points), and teamwork (5 points), with a total score of 100 points. After the assessment, the teacher will take the average of the total scores of the group as the final assessment result of the workshop.

#### Innovation achievement index

After the completion of the teaching activities of the innovation practice workshop, the staff will follow up on the feedback of the innovative scientific research results for one year for each group of participants and count the number of scientific research results such as patent application, patent authorization and thesis writing for each group as the main innovation results index.

### Statistical methods

The SPSS 25.0 software was used to establish the database for data entry and statistical analysis, in which the count data were statistically described by frequency and composition ratio, and the measurement data were described by X ± S. Two independent samples t-test was used to compare the results of creativity scale scores and presentation practice assessment results between two groups; the rank sum test was used to compare the results of teaching satisfaction, and P < 0.05 indicated that the difference was statistically significant.

### Ethical approval

We designed and conducted the study following the guidelines for ethical principles outlined in the Declaration of Helsinki. All methods carried out were by West China Second University Hospital, Sichuan University. The participants were informed about their anonymity, their right to withdraw from the research, and the voluntary nature of the study. Informed consent was obtained from the participants at all stages of the study.

**Table 1:** Teaching design of nursing innovation practice workshop

Theoretical guidance		Course Sessions	Class Type	Teaching method	General content
Task Related Motivation	Questioning and Preparation Stage	Before Class Reflections and Preparation	Self Study	Online	A pre-class reflection task is issued by the instructor to inspire participants to identify problems in the clinic, such as inconvenient instruments, equipment, tools, and procedures that may exist in the clinic. The participants are guided to think and are asked to develop a preliminary idea of the problem they have identified by conducting a data search through their own thinking and literature search.
Domain Related Skills	Preparation Stage	Theory Lectures	Teaching	Offline	Theoretical lectures included: ① overview and significance of nursing innovation; ② overview of patents (role, types, characteristics, significance, etc.); ③ trends related to patents, conditions for granting, and methods of application procedures; ④ methods of searching patents; ⑤ methods of nursing innovation; ⑥ how to practice nursing innovation and other aspects of theoretical courses.
		Case Lectures	Cases Studies	Offline	Patents on nursing/medical consumables, appliances, instruments, and equipment that are outstanding in terms of innovation, practicality, scientificity, and operability are selected as cases, and combined with the speaker’s nursing innovation experience, the technical field, background technology, innovation direction, patent content and specific implementation of the patents are elaborated.
Domain Related Sskills	Practice and Validation Stage	Workshops Practice	Discussion	Offline	The lecturer asks the following questions: “In your usual clinical work or operation, do you encounter any inconvenient and inconvenient times?“; “If you were to improve this inconvenient situation, what would you do to improve it? If you were asked to improve this inconvenient situation, how would you do it?“ The participants will be inspired and guided by “thinking about whether there are any innovative (improvement or creation) ideas for practical clinical problems in their daily work”, as well as by intra-group discussions, in which each group member will actively express their ideas, briefly describe the problem, background, innovative ideas, and solutions. The group selects the best idea for the group; then the group brainstorms and discusses in depth the direction
Individuals Have the Motivation			Practice	Offline	After completing the discussion, the group unified their opinions and completed the first draft of the “nursing innovation proposal” according to the framework of the “nursing innovation proposal” (including name, technical field, background technology, purpose and significance, technical solution, beneficial effect, innovation point, social value, economic value, and attached figure).
	Evaluation Stage	Program Showcase	Reporting	Offline	The group leader will be responsible for the presentation of the group’s “nursing innovation”. Each group will be presented in 5–10 groups, including the background of the nursing innovation, the expected goal, improvement/solution, beneficial effect, innovation point, social benefit, economic benefit and sketch, and the specific division of labor within the group. The teacher will also score the presentation and give evaluations and suggestions.
	Continuing and Output Stage	Improvements and Outputs	Tracking	Online + Offline	At this stage, the faculty member acts as a mentor to guide the process and track the results for one year, guiding each group’s innovative proposal and giving necessary assistance throughout the process. The team leader of each group is responsible for organizing the team members to work together in class, and then revising and refining the first draft of the “nursing innovation plan” through data review, patent search, and working with the patent staff to form a “patent application” and submit it to The patent application was submitted to the National Patent Office.

## Results

### General information

A total of 50 participants were included in the study, including 5 males and 45 females; mean age 21.66 ± 0.80 years; 31 with college degrees and 19 with bachelor’s degrees. The study population was randomly divided into 12 groups for teaching activities.

### Results of creativity scale scores

In terms of the creativity scale scores before and after the implementation of the curriculum, the creativity scale scores after the implementation of the curriculum (23.26 ± 3.16), the results are higher than the results of scores before the implementation of the curriculum (19.98 ± 4.62), the difference is statistically significant (t = 4.144, p < 0.001), the detailed results are shown in Table 2.

### Teaching satisfaction results

According to the results of the satisfaction of this teaching, 47 out of 50 people said they were very satisfied and 3 people said they were satisfied, so the satisfaction of teaching (47 + 3)/50*100%=100%. In addition, 50 out of 50 people (100%) who participated in this course said they were willing to participate in this course again.

### Results of the practical demonstration assessment

In the results of practical assessment of the workshop presentation, the final average score of each group presentation (82.25 ± 10.21), and the scores of other dimensions are detailed in Table 3.

### Number of research results

After the course, the participants were tracked for one year, and the tracked research results included patent application, patent grants, and paper writing related to this innovation course; the results showed that the total number of research results was 19, as shown in Table 4; moreover, the patent application rate of the participants reached (12/12*100%=100%), which successfully realized the transformation path from research thinking to patent application. 

**Table 2:** Comparison of the results of creativity scale scores before and after the implementation of the curriculum

Course Implementation	N	Propose original and relevant research questions	Suggestions for interpreting research questions from a new perspective	Invention or ingenious use of existing research techniques, research tools and experimental equipment	Propose new paths, methods, processes, and tools to break through research dilemmas	Discovering new evidence to answer research questions in the course of research work	Summarize research findings and propose new theories that can be generalized	Total Score
Before	50	3.62 ± 0.92	3.34 ± 0.85	3.34 ± 0.82	3.30 ± 0.84	3.36 ± 0.85	3.02 ± 0.82	19.98 ± 4.62
After	50	4.44 ± 0.61	4.16 ± 0.58	3.86 ± 0.61	3.96 ± 0.60	3.64 ± 0.69	3.20 ± 0.78	23.26 ± 3.16
T value		5.236	5.632	3.596	4.512	1.804	1.123	4.144
P value		<0.001	<0.001	<0.01	<0.001	0.074	0.264	<0.001

**Table 3:** Assessment results of workshop practice demonstration

Dimensions	Full score	Scores
Science of the program	15	11.7 ± 1.84
Innovation of the program	15	11.95 ± 1.79
Practicality of the program	15	12.45 ± 1.57
Operability of the program	15	11.85 ± 2.02
Content and form of presentation	20	17.2 ± 1.46
Information Retrieval and Use	10	8.1 ± 1.35
Language expression and communication	5	4.7 ± 0.42
Teamwork	5	4.55 ± 0.64
Total Score	100	82.25 ± 10.21

**Table 4:** Number of scientific results for a one-year follow-up period

Item	Number of groups	Patent Application	Patent Grant	Paper Writing	Research Project	Honor and Award	Outcome Total
Total	12	12	4	1	1	1	19

## Discussion

### The innovation practice workshop model under the OBE concept contributes to the improvement of innovation ability and the output of research results

The data presented in Table 2 highlights a marked increase in participants’ scores on the creativity scale post the adoption of the innovation practice workshop model under the OBE concept, recording 23.26 ± 3.16, compared to pre-implementation scores of 19.98 ± 4.62. This variation was statistically significant with t = 4.144,p < 0.001. Such an outcome can likely be attributed to the instructional design structured around the creativity component theory and an outcome-driven workshop model emphasizing experiential, participatory, and interactive elements. Lectures, based on this model, guide participants progressively through the creative process, leveraging case studies for layered analyses, which foster creative potential exploration. Group discussions bolster individual learning and reciprocally enhance peer learning [25]. With facilitator and peer interactions, participants incrementally refine their perspectives, reinforcing knowledge construction. Emphasis on critical thinking throughout lectures cultivates active and dialectical thought processes, steering participants through comprehensive problem-solving stages: problem identification, analysis framework construction, key determinants, efficient execution, and iterative evaluations.

During instructional sessions, educators meticulously steer participants through the innovation journey, promoting active participation and thought, thereby unlocking their creative potential. Collaborative exercises with diverse departmental representatives in each group lead to multifaceted brainstorming. Interdisciplinary discourse offers a rich tapestry of perspectives, thereby refining and diversifying solutions. Such effective intra-group communication not only reinforces teamwork and collective ethos but also refines independent learning capacities, problem-solving aptitudes, and literature review skills, eventually enhancing professional self-efficacy and paving the way for career progression. With educators’ guidance in practical sessions, participants receive timely feedback, fostering educator-participant rapport and unearthing deeper insights [26, 27].

Regarding research output, a one-year follow-up (Table 4) revealed 19 distinct research outcomes, encompassing patent applications, patent grant, paper writing, research project, hornor and award. The innovation workshop, based on the OBE concept, seems propitious for tangible research outcomes. Most novice nurses, being recent graduates, bring a fresh perspective, still untethered to prolonged clinical practices. Certain hospitals employ multidisciplinary rotations to nurture multifaceted clinical expertise. The objective being to reinforce clinical knowledge, hone clinical skills, and broaden clinical perspectives. During such rotations, timely stimulation of innovative thinking, complemented by guided innovation practice, better equips nurses to actively confront clinical challenges, delving into potential solutions.

### The innovative practice workshop model under the OBE concept helps to improve the teaching effectiveness and satisfaction of teaching

A satisfaction survey revealed 100% satisfaction among participants, with 47 rating the course highly satisfactory. This stands in stark contrast to last year’s course, which registered 78.0% satisfaction. Furthermore, all attendees expressed eagerness to re-enroll. Participant testimonials illustrated profound transformations in their perception of innovation and self-worth. When conducting the teaching situation interviews with the participants, some participants said, “I never thought I would become an inventor”, “I did not think I would have the ability and opportunity to make inventions”, “I have a new I have a new understanding of my abilities”, “there are many places to innovate in my work”, “I feel the collision of ideas in different departments”, “I know a lot of new knowledge”, etc. The course was a great success. In summary, the participants not only learned about innovation during the course but also gained a deeper understanding of innovation. In practice, they experience the process of complete creation, improve their ability to discover, analyze and solve problems, and acquire innovation-related skills, while reaping the results of scientific research, which is positive and timely positive feedback. This allows participants to not only gain knowledge and skill level improves, but also a sense of accomplishment, satisfaction and achieving tangible research results.

Regarding teaching efficacy, the average assessment score for participants’ presentations stood at 82.25 ± 10.21. In these five aspects have achieved a high score: the program’s science innovation, practicality, operability, the content and presentation format, and the information retrieval and use. These outcomes likely stem from the course’s design, which integrates the creativity component theory and emphasizes the five pillars of creativity: questioning, preparation, practice, verification, and evaluation. Participants were grouped prior to the course initiation, and the educator posed thought-provoking questions, affording participants ample contemplation time and access to diverse resources. This approach set the stage for deriving solutions that were scientific, innovative, practical, and actionable. Concurrently, the course guided participants in transitioning from abstract contemplation to tangible insights, enabling them to identify pivotal aspects of creative conception and innovation. Such an approach fostered a concentrated emphasis on pivotal areas, facilitating precise advancements in subsequent patent designs. As a result, the innovation strategies emerged more distinct, streamlined, and structurally refined.

## Conclusion

In summation, the innovation workshop tailored for nurses, grounded in the creativity component theory and the OBE concept, elevates teaching satisfaction and efficacy. It seamlessly fuses theoretical knowledge with practical application, stimulating participants’ cognitive engagement and enthusiasm. This approach amplifies participants’ proactive contributions, fostering research output. Throughout the teaching sessions, participants enhance their creative and problem-solving capacities, alongside fostering teamwork, literature analysis, and communication proficiencies. Such skill augmentation not only bolsters their overall competencies but also paves the way for future career growth. As the pursuit of nurturing innovative talent within nursing continues, it beckons further comprehensive investigation.

## Limitations

The present study employed four evaluative metrics: creativity scores, teaching satisfaction levels, research outputs tracked over a year post-course, and workshop presentation evaluations. Future research could enrich objective criteria, prolong the observation period, and broaden assessments to encapsulate various facets of practice, exploration, and efficacy. Furthermore, fostering innovative thought and aptitude hinges on the harmonious blend of theoretical instruction and hands-on experience, complemented by sustained innovation exercises in subsequent stages. Identifying the optimal approach to cultivate innovative nursing talents remains a salient focus for ensuing studies.

## Data Availability

The datasets generated and/or analysed during the current study are not publicly available due to the containing information that could compromise the privacy of the research participants but are available from the corresponding author on reasonable request.
